# The Influence of Interdisciplinary Work towards Advancing Knowledge on Human Liver Physiology

**DOI:** 10.3390/cells11223696

**Published:** 2022-11-21

**Authors:** Blanca Delgado-Coello, Nalu Navarro-Alvarez, Jaime Mas-Oliva

**Affiliations:** 1 Department of Structural Biology and Biochemistry, Instituto de Fisiología Celular, Universidad Nacional Autónoma de México, Mexico City 04510, Mexico; 2 Department of Gastroenterology, Instituto Nacional de Ciencias Médicas y Nutrición Salvador Zubirán, Mexico City 14080, Mexico; 3 Departament of Molecular Biology, Universidad Panamericana School of Medicine, Mexico City 03920, Mexico; 4 Department of Surgery, University of Colorado Anschutz Medical Campus, Denver, CO 80045, USA

**Keywords:** liver regeneration, compensatory hyperplasia, interdisciplinary research, tissue engineering, decellularization, xenotransplantation

## Abstract

The knowledge accumulated throughout the years about liver regeneration has allowed a better understanding of normal liver physiology, by reconstructing the sequence of steps that this organ follows when it must rebuild itself after being injured. The scientific community has used several interdisciplinary approaches searching to improve liver regeneration and, therefore, human health. Here, we provide a brief history of the milestones that have advanced liver surgery, and review some of the new insights offered by the interdisciplinary work using animals, in vitro models, tissue engineering, or mathematical models to help advance the knowledge on liver regeneration. We also present several of the main approaches currently available aiming at providing liver support and overcoming organ shortage and we conclude with some of the challenges found in clinical practice and the ethical issues that have concomitantly emerged with the use of those approaches.

## 1. Introduction

Liver regeneration was implicitly recognized in the Greek Prometheus and Tityus myths, but it was Carl von Lagenbunch who performed the first documented successful hepatectomy in humans in 1888 [1,2]. Since then, the interdisciplinary character of scientific progress has been evident. For instance, the observations made by the chemist Louis Pasteur, who for the first time recognized the close relationship between microbes and infectious diseases, were successfully interpreted by the British surgeon Joseph Lister in 1865. This knowledge led him to introduce the use of diluted solutions of carbolic acid as an antiseptic agent [3].

**Figure 1:**
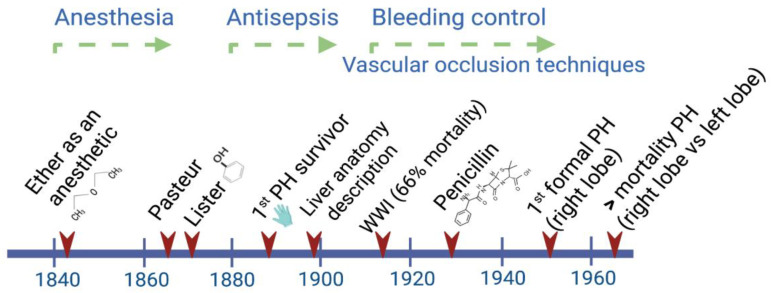
Main advances through the early history of liver surgery have involved interdisciplinary work. By the time Lagenbunch managed to achieve patient survival following a partial hepatectomy (PH), antiseptic procedures including the use of gloves resulted in a greater chance of success. At the beginning of the modern period after World War II, the better control of hemorrhages significantly contributed to a reduction in the mortality rate after right/left liver resections [4].

During the period between 1880 and World War II, a big step forward in attaining better survival rates was achieved by the implementation of antiseptic procedures during and after any kind of surgery. After World War II, the main efforts were focused on deepening the knowledge of liver anatomy and the development of different technologies to provide liver support to patients (Figure 1).

Unfortunately, in the last few decades, important challenges directly affecting the liver have arisen related to modern lifestyles, such as poor eating habits, low physical activity, and environmental factors, which have skyrocketed the incidence of liver diseases. A possible option to overcome the pace at which liver diseases are increasing is to take advantage of the regeneration capacity the liver inherits, which can only be achieved by gaining a better and deeper understanding of this important process.

In this review, we describe the interdisciplinary research involved in the study of liver regeneration associated with surgical resection, and its impact on the current knowledge of this process in humans. First, we include some of the contributions obtained from interdisciplinary work that are applied to human physiology either using classic animal models or mathematical models to understand and predict possible outcomes following hepatectomies. Then, considering that the main challenge continues to be achieving an optimal recovery of the liver tissue, especially when severe chemical or physical damage threaten the physiological capacity of the liver, we explore recent insights in the fields of tissue engineering and xenotransplant. Finally, we expose some ideas regarding the many ethical issues that should be considered when research involves the use of animal models and their application in humans.

## 2. Interdisciplinary Models Applied to Translational Medicine

### 2.1. Animal Models of Liver Regeneration

Even though the regenerative capacity of the human liver has been long recognized, the use of animal models has played a key role towards advancing clinical practice. Rat and mouse models are the most widely used due to their size, costs, reproducibility, and ethical advantages.

Most of the knowledge related to liver regeneration in mammals has been obtained from the classical model described in the rat, known as 70% partial hepatectomy (PH), which involves the resection of two thirds of the liver tissue [5]. At that time, although the mechanisms were not understood, it was known that cell division in the hepatic tissue was a rare event that might be reverted by performing a hepatectomy. The first attempts to understand this process came from experiments using the parabiosis approach, where two or three animals shared blood circulation. It was demonstrated that, after performing 70% PH in one of the two partners (or after 80% PH in two of three rats), the intact liver showed high mitosis rates, and an increase in liver weight and in the number of total cells [6,7]. These findings supported the suspected role played by the portal blood flow in the liver and prompted the search for the specific humoral factors involved.

A constant widely documented in mammals is the strict proportionality of around 3% between the liver mass and the individual’s size. For example, when a large dog receives a liver from a smaller dog, the transplanted liver grows to reach the required mass [8]. Accordingly, liver size increased after a patient was transplanted with the liver of a baboon, although he only survived for 70 days [9]. This observation led to the concept of “hepatostat” in reference to the programmed stability of the liver size that is maintained by the complex sensing and signaling molecular toolkit that starts a few minutes after a liver section is resected and continues until the original liver mass is recovered [10]. Therefore, liver regeneration is now strictly conceived as a compensatory hyperplasia process where the lost mass is replaced by the remaining tissue.

Regarding the mechanisms involved in the process of liver mass recovery, Higgins and Anderson observed an increase in cell size (hypertrophy) as an early response followed by mitosis two or three days after PH [5]. Later, these processes were further studied in mice, and now we know that hypertrophy alone may explain regeneration when 30% of the liver is resected; in contrast, when 70% PH is performed, hypertrophy and the increase in the number of cells (hyperplasia) are involved [11,12].

One of the main contributions provided by research in small species has been the use of transgenic and knockout mouse models; this topic has been extensively studied by Fausto’s group and recently reviewed by other research groups [13,14,15]. Together, these studies have shown that physiological factors such as cytokines and other signaling molecules regulate liver regeneration. It has also been demonstrated that when some of the genes regulating these factors are knocked out, liver regeneration still progresses although at a lower rate, indicating the compensatory mechanisms performed by the diverse molecules.

In addition, by using lineage-tracing techniques and the analysis of functional or metabolic markers, it is now known which specific population of hepatocytes contributes to hepatic renewal after PH, and the possible origin of cells differentiating into hepatocytes [16,17,18]. Interestingly, the distribution of tasks in the liver lobules seems to contribute to proper functioning, even while liver regeneration is taking place.

While the regeneration process is similar between small mammal species and the human [19], larger experimental animals offer easier handling and a closer proximity to human physiology. Therefore, liver resection at different extents has been performed in other species such as dogs [20], pigs [21], and non-human primates [22]. Pigs, with similarities to humans regarding feeding habits, anatomy, and physiology, have become a good model to study liver regeneration, and to explore one of the most common complications following liver resection, which is the development of small-for-size syndrome (SFSS) [23,24,25,26]. This syndrome is triggered by excessive portal venous inflow and the small remnant liver, resulting in risks such as cholestasis, liver insufficiency, and even death.

Liver regeneration involves complex mechanisms that take place along three stages [10,27,28,29]: (1) the priming phase, where proinflammatory cytokines (TNF-α and IL-6) prepare the cells to enter the cell cycle; (2) the proliferative phase, where the activation of several signaling pathways (JAK/STAT, MAPK, and PI3/AKT) promotes mitogenesis [30,31]; and (3) the termination phase mediated by inhibitory cytokines of the TGF-β superfamily, triggering the transcription of genes to stop growth and return the liver tissue to the quiescent stage [32]. When people donate a part of their liver (or receive it), the liver tissue reaches ~80% of its original mass after six weeks, and the total recovery of the liver tissue takes about six months. This is accomplished by a typical liver regeneration mechanism that depends on pre-existing hepatocytes that replicate one day after PH [33]. After surgical resection, all the cell types of the liver are activated. Then, after receiving signals from the complement system, Kupffer cells release the proinflammatory cytokines TNF-α and IL-6 that, through paracrine communication, prime hepatocytes by activating specific transcription factors. Meanwhile, hepatic stellate cells secrete the inactive hepatocyte growth factor (HGF) that is extracellularly activated and reaches its receptor in the hepatocyte. At that point, hepatocytes turn responsive to growth factors such as the epidermal growth factor (EGF) and the transforming growth factor α (TGF-α), and enter the G1 phase of the cell cycle. Proliferating hepatocytes produce growth factors and stimulate mitosis in other cell types. In humans, after the replication of different hepatic cells, a hypertrophic mechanism restores the liver volume [34], then the cytokines of the TGF-β superfamily, produced by hepatic stellate cells and Kupffer cells, control the termination phase indicating that proliferation should stop [33,34].

**Figure 2:**
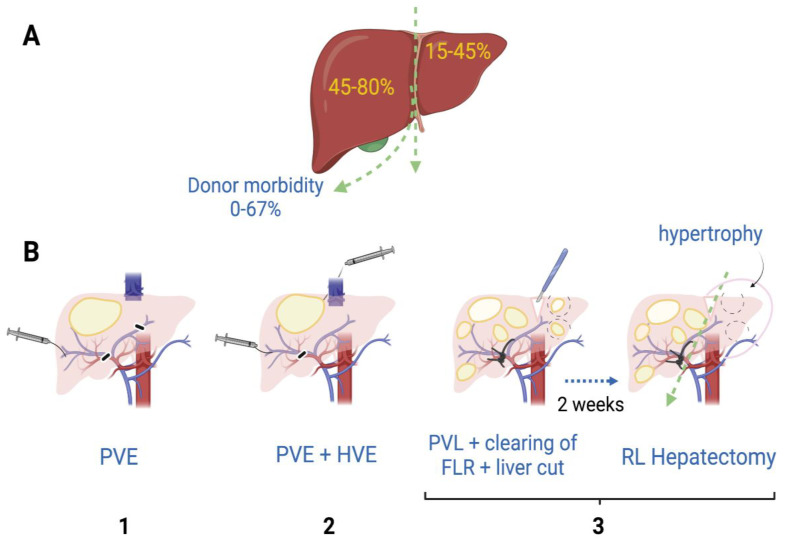
Hepatectomy of the right lobe is implicitly riskier. (**A**) The right lobe represents, on average in healthy humans, 45–80% of the whole liver volume [37], while morbidity of right lobe donors is estimated to be 0–67% [38]. (**B**) When the right lobe is compromised for the presence of one or more tumors, patients might need extensive hepatectomy. To minimize small-for-size syndrome risks and promote hypertrophy of the left lobe, liver surgeons have practiced some of these procedures previous to the right lobe hepatectomy: (1) Right portal vein embolization (PVE) by introducing occlusive materials (e.g., ethanol, microparticles); (2) Simultaneous embolization of portal and hepatic veins (HVE); (3) In cases where left lobe also has tumors, portal vein ligation (PVL) together with ablation of left lobe tumors (clearing of future liver remnant; FLR) and liver transection have been performed. After 1–2 weeks, once left lobe shows hypertrophy, a right lobe (RL) hepatectomy can be performed [35].

Supported by the vast knowledge acquired throughout the years about liver regeneration and the improvement in surgical techniques, extensive hepatectomies are currently performed in patients with advanced liver cancer. In extreme cases, where surgeons anticipate that the size of the liver after resection will be too small, they have the ability to perform right portal vein embolization or ligation alone among other procedures, in order to promote liver parenchyma augmentation through a hypertrophic mechanism [35] (Figure 2B). These techniques have been developed based on the observation made a century ago, when it was recognized that the occlusion of a segment of the portal vein or the hepatic artery of the rabbit produces hypertrophy in the contralateral lobe while atrophy occurs in the side of occlusion [36]. Together, portal and hepatic vein embolization induce faster growth of the contralateral liver than portal vein embolization alone [35].

**Figure 3:**
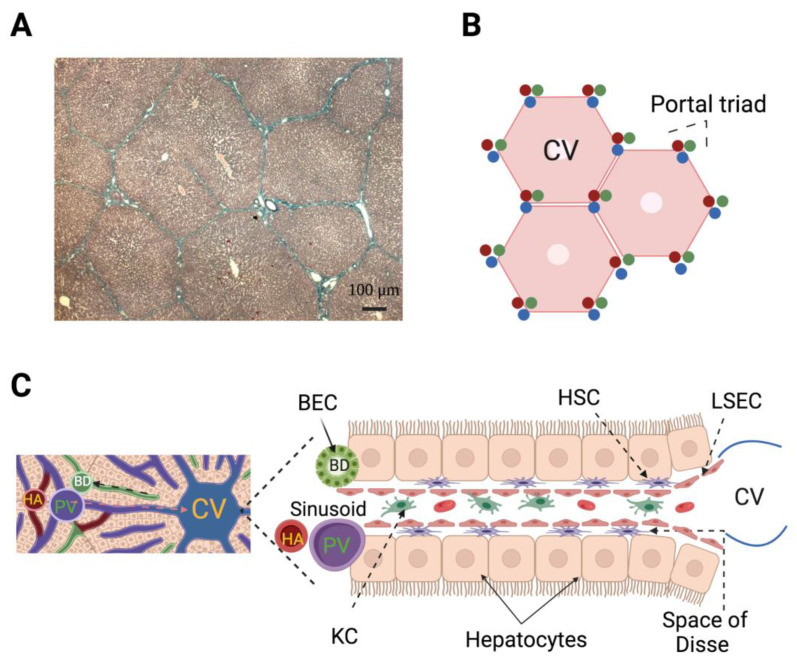
Hepatic lobules represent the functional unit of the liver. (**A**) Masson trichrome-stained histological section showing the polygonal shape of hepatic lobules from pig (4X). (**B**) Representation of the human hepatic lobule according to the vascular network model based on fluid mechanics where the hexagonal shape was determined to be optimal to avoid pressure losses because flow resistances are minimal. Note that each portal triad makes contact with three lobules. (**C**) Left: Liver lobule close-up; the direction of blood (orange arrow) and bile (black arrow) flows are indicated. Right: Hepatic microcirculation composed of capillaries (sinusoids) lined by liver sinusoidal endothelial cells (LSEC) where blood flows of the hepatic artery (HA) and portal vein (PV) converge. In the sinusoid, Kupffer cells (KC; resident macrophages of the liver) protect the liver from pathogens and produce proinflammatory cytokines after PH; KC coexist with different types of lymphocytes. On each side of the sinusoid the hepatocytes (parenchymal cells) are linearly distributed leaving a space between them and the sinusoid wall—the space of Disse—where hepatic stellate cells (HSC) are located. The HSC under quiescent conditions store droplets full of vitamin A, after PH become activated, then the droplets are depleted, cells change their phenotype to a myofibroblast-like one, and contribute to regeneration at different stages. The bile duct (BD) is composed by biliary epithelial cells (BEC, or cholangiocytes). The BEC share with hepatocytes the embryonic lineage from hepatoblasts, and importantly, under extreme conditions BEC dedifferentiate to liver progenitor cells, which in turn differentiate into hepatocytes [18]. The LSEC, KC, HSC, BEC, and lymphocytes are included in the group of non-parenchymal cells.

The mechanisms mediating the regeneration process, after performing these procedures followed by a subsequent hepatectomy, have been an area of intensive research. Despite important hemodynamic differences such as the preservation of the arterial flow in the case of embolization, the mechanisms for liver regeneration between portal vein embolization and partial hepatectomy are believed to be essentially the same [39]. However, there have been some controversies, since based on the observation that the administration of follistatin, an activin inhibitor, did not increase regeneration in non-ligated lobes, it has been suggested that different mechanisms might be involved [40]. What is clearly known is that the hemodynamic changes caused by the alterations in portal flow are related to the hypertrophy of the liver [41]. In the case of the Associating Liver Partition and Portal Vein Ligation for Stage Hepatectomy or ALPPS procedure (Figure 2B-3), it has been observed that hepatocytes show higher proliferation potential when compared to portal vein ligation alone, which is believed to be potentiated by the soluble growth factors and cytokines released when performing the liver resection [42]. The hepatocytes are crucial to accomplish liver mass restoration and similar signaling pathways are involved, but it seems that mainly immature cells participate after ALPPS, while mature cells are involved in regeneration after portal vein embolization [43]. The role of non-parenchymal cells under these conditions is still unknown.

It should be noted that in humans, liver regeneration can also be triggered by chemical agents; however, the stages by which the process takes place have been described based on the PH model [14]. Moreover, liver resection is also preferred because it does not involve necrotic damage, which may cause an additional inflammatory stimulus that may turn detrimental for the regeneration process.

Together, this knowledge supports the notion that liver regeneration evolved as an adaptive response to liver injury [44]. Regardless of their size, all known vertebrate models have contributed to advance the knowledge related to liver regeneration, starting with the zebrafish, which shares not only a high proportion of orthologous genes with the human, but some of the cell plasticity mechanisms that the liver displays after severe damage [45].

### 2.2. Models Applied to Predict Different Scenarios after Liver Resection in Humans

The liver receives a double blood supply, 75% coming from the portal vein (deoxygenated blood) and the rest provided by the hepatic artery (oxygenated blood). Once inside the liver, the portal vein and the hepatic artery branch irrigate the lobules that are the functional unit of the liver. The lobules have a polygonal shape, wherein their vertices converge small branches of the portal vein, the hepatic artery, and the bile duct; these three components make the portal triad. The blood flow follows through the sinusoids toward the central vein in the middle of the lobule, while the bile flow runs in the opposite direction through the canaliculi of the hepatocytes (Figure 3).

The liver depends strongly on its vascular system to trigger early responses to stimuli such as 70% PH, after which the remnant liver abruptly receives the blood supply originally needed for a complete liver; thus, the portal flux for the remnant liver is estimated to increase three-fold [46]. Accordingly, the factors promoting liver regeneration coming from the portal vein are also concentrated. Associated with this phenomenon, there are several clinical risks observed in patients after receiving a transplant such as post-hepatectomy liver failure (PHLF) and the activation of small-for-size syndrome (SFSS). Motivated by these concerns, several interdisciplinary groups have worked on developing not only formulas to estimate the remnant liver mass after the partial resection of the liver in humans and pigs [47,48], but also models to understand the normal physiology of the liver vasculature and to predict scenarios in patients after undergoing surgery. Interestingly, based on data obtained from donors that had undergone right lobe hepatectomy, the adverse effects on donor outcomes were estimated to occur when the ratio of remnant liver/total liver volume was ≤30% [37].

There have been several other models focused on reducing the risks of liver failure at the cellular, lobular, organ, and the whole-body scale; a recent comprehensive review addresses these topics [48]. Here, we review several works focused on the lobular scale.

The availability of information (e.g., 3D-CT angiographies and computed tomography scans) obtained from the donors and recipients of liver transplants has contributed to make important clinical predictions. For example, the recovery time needed for liver donors has been estimated using a model based on the metabolic load imposed by liver regeneration [49]. Also, another research group interested in preventing post-hepatectomy liver failure validated and reported a model that predicted the probability of success of a liver resection [50].

The vascular network of the human liver has been modeled and it has been determined that all vessels overlap and have a predictable dendritic nature [51,52,53]. This pattern is an efficient system to irrigate the whole lobule, and its configuration has been demonstrated to be optimal at reducing pressure losses (Figure 3B). This model was taken further by considering a total number of lobules close to 4.8 × 10^6^, and other parameters associated with each lobule: volume, length of one side of the hexagon, thickness, radius, permeability, and mass flow rates and internal/outer pressures [54]. Then, the authors simulated conditions where several hepatic lobules were “obstructed” to reach theoretical liver resections of 33 or 78%. They observed increases in portal pressure and portal flow, and a decrease in arterial flow with no change of hepatic arterial pressure, mimicking the causes of SFSS [54].

These models represent a valuable interdisciplinary tool, since they are based on liver parameters observed in humans, and therefore provide a good risk estimation that can help doctors in decision-making.

## 3. Strategies to Face Current Challenges in Clinical Practice

### 3.1. Extracorporeal Liver Support and Liver Preservation Techniques

**Figure 4:**
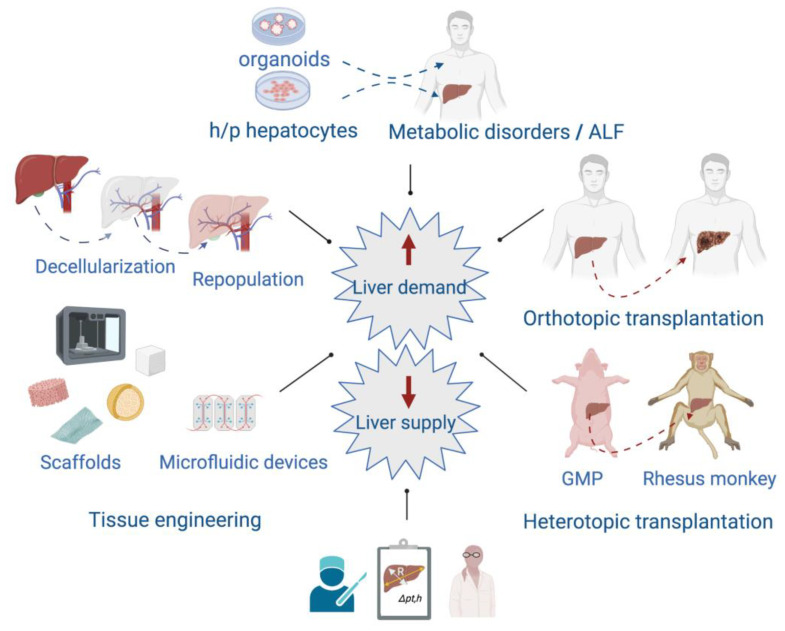
Interdisciplinary approaches focused on providing alternatives to patients affected by acute liver failure (ALF) and end-stage chronic liver diseases. In the left panel, some of the tissue-engineering techniques applied to provide scaffolds of different types are shown. At the top are included the transplantation of organoids or human/pig (h/p) hepatocytes to treat acute liver failure (ALF) and metabolic disorders. The right panel shows the orthotopic liver transplantation between individuals of the same species, and the heterotopic transplantation that involves donation from one species to another. Heterotopic transplantation can overcome the human organ shortage although there are several issues to solve such as hyperacute rejection and coagulation dysregulation. Nowadays, the use of genetically modified pigs (GMP) and immunosuppressive therapy are improving the recipients’ survival and increasing the possibility of eventual use in humans. At the bottom, the development of mathematical models as helpful tools in decision-making to perform liver surgeries.

Although the liver has a powerful capacity to regenerate, there are several end-stage chronic liver diseases such as cirrhoses of different etiology, hepatocellular carcinomas, and some severe acute liver diseases that go beyond this ability. In these cases, patients may require a liver transplant from a living or a deceased donor. Unfortunately, the number of people needing a liver transplant is far higher than the number of donors [55]. To face these challenges, the scientific community has implemented different interdisciplinary strategies attempting to provide temporal support to patients or long-term solutions to pathologies affecting the liver, and to prevent the risks associated with extended hepatectomies (Figure 4).

Currently, several devices are available that can provide temporal liver support to patients while waiting for a liver transplant (bridging transplantation) [56]. These devices perform the functions of detoxification, regulation, and synthesis, contributing to improve the survival of patients with acute liver failure [57,58,59]. These devices have been tested as a therapeutic bridge while the liver regenerates or while an organ becomes available. These detoxification systems can utilize living cells or are cell-free systems that use albumin mainly as a detoxifying agent [59,60]. There have been contrasting results, with some indicating their benefit while others have shown no benefit [61]. The benefits observed have been mainly related to the improvement in biochemical parameters including bilirubin, ammonia, creatinine levels, and inflammatory cytokines among others, and consequently a benefit in encephalopathy [57,62,63]. Although the benefit in survival has been limited, research in this field continues due to the promising potential these devices have [59].

On the other hand, given the imbalance between the high demand for a liver for transplantation and the actual liver donors, the requirements to donate have been expanded according to the “extended criteria donor”, where age and the presence of fatty liver are no longer used as exclusion parameters. Under these criteria, a few years ago an ex situ procedure consisting in the dynamic preservation of the liver previous to transplantation was adopted [64]. To date, it is possible using a normothermic machine perfusion to preserve the liver for >24 h up to 7 days. This automated machine works at 37 °C and attempts to mimic a more physiological scenario by pumping solutions and employing oxygenating systems recreating what the heart and lungs would do in the body. This machine also removes metabolic waste as the kidneys do, and by adding hormones and nutrients the functions of the intestine and pancreas are mimicked [65]. The use of a hypothermic machine (4–6 °C) has also been shown to reduce very common ischemia-reperfusion injury. Machine perfusion systems have also been used for the testing of different therapeutic components to be added to the perfusate in order to improve liver quality, aiming at increasing transplantation success. However, there are still some issues to be solved such as the development of new viability criteria, as well as the assessment of the safety and efficacy of this method.

### 3.2. Tissue Engineering Techniques and Biotechnological Advances

Scaffolds made of different biocompatible materials (synthetic or natural), which provide conditions similar to the native extracellular matrix (ECM) of the liver, have helped advance the knowledge of liver regeneration [66,67]. The decellularization of the liver, for instance, provides a scaffold that maintains the native ECM and the hepatic vasculature that could be used in repopulation experiments to study liver regeneration.

The best procedure for whole-liver decellularization uses the perfusion of detergents, enzymes, and chelating agents through the portal vein that solubilize lipids and eliminate cells and nucleic acids. In order to preserve the 3D ultrastructure, composition, and biological activity of the ECM, the procedure needs to be optimized by using different concentrations, combinations of reagents, and time and pressure used for perfusion. After the whole procedure, a washing step to eliminate the decellularization reagents and sterilization with gamma radiation may be needed to reseed the organ. The first attempts to achieve liver decellularization were performed in small species; later, the procedure was reported in pigs, sheep, rabbits, and in humans [68,69,70,71].

There has been some success in humans using this procedure; Mazza et al. performed perfusion of the left lobe over 14 days, while the whole human liver took 6 weeks to be completely decellularized [71]. They reported a good preservation of the liver tissue architecture, where collagen types I, III, IV, and fibronectin were also detected. Importantly, small cubes obtained from the whole decellularized liver were seeded with different human cells (hepatic stellate cells -LX2-, HepG2, and SK-Hep1 cells) that showed growth for 21 days [71]. Additional studies have shown well-preserved ECM by histology, electron microscopy, and proteomic analyses and successful repopulation with mesenchymal stromal cells or endothelial cells from the umbilical vein that restored the vascular lining [72].

The final goal pursued by the decellularization of the liver is to obtain a “bioengineered liver” that can be transplanted and relieve the shortage of organs available for transplantation. Once the liver is cell-free and the ECM is well preserved, a bioengineered liver must accomplish the following properties: a proper vascular permeability to distribute new cells in the right location, reseeded cells that can be of parenchymal (hepatocytes) or non-parenchymal type (e.g., hepatic stellate cells, endothelial cells), and the provision of oxygen and nutrients supporting cell viability. Although scientists have implemented several strategies to solve these issues, to date only short graft survival has been reached [73]. The additional technical problems that are still under intense research are those related to maintaining hepatocytes in a differentiated state. The use of growth factors and culture conditions, including the use of collagen-I, have mildly alleviated this issue. The availability of hepatocytes for their direct transplantation or for the reseeding of the decellularized liver is also still a limitation that has prompted the use of embryonic stem cells. An important contribution made by Takahashi and Yamanaka in 2006 consisted in the use of induced pluripotent stem cells (iPSCs) obtained from reprogrammed mouse embryonic or adult fibroblasts avoiding the immunological rejection associated with stem cells of embryonic origin [74]. This procedure was then successfully reproduced using adult human fibroblasts [75]. Pluripotent stem cells (PSCs) have significantly contributed to improve our understanding of hepatocyte differentiation and this knowledge has been applied towards the development of 3D models, such as spheroids and organoids, aiming at better mimicking the liver structure. Important contributions have been made by Takebe et al., who used different cell types to produce hepatocytes and an arrangement similar to the liver tissue [76,77]. Mun et al. have also shown the formation of hepatocyte-like liver organoids from PSCs including embryonic stem cells and iPSCs. These organoids expressed mature hepatocyte markers and thus the ability to produce proteins such as albumin and metabolized drugs, therefore representing an important tool for toxicological outcome production [78]. Moreover, these organoids allowed their use to test and study the regenerative capacity of the liver after chemical insults such as the administration of acetaminophen, making these models an extremely important tool for studying liver regeneration, modeling liver disease, drug screening, and for personalized medicine [78,79,80,81,82]. With the most recent advances in the field, it is now possible to produce functional liver spheres by the self-aggregation of hepatocytes, endothelial cells, and hepatic stellate cells properly differentiated from human pluripotent stem cells (hPSC) [83]. Moreover, these procedures have been automated with excellent results in terms of functionality, reliability, and reproducibility [83]. Additionally, and importantly, there are currently optimized protocols to obtain hepatocytes from PSCs, reducing the risk of teratoma formation due to the presence of remnant undifferentiated PSCs [84,85]. Interestingly, the known liver zonation associated with the O_2_ gradients that determine functional and metabolic differences within the liver [86] has also been achieved in the liver spheres, which has supported mathematical models that determine in advance the relevance of performing experimental work [87].

On the other hand, recently, an in vitro model developed to deepen the knowledge of liver physiology in humans was achieved by employing a one-channel microfluidic device. In this model, potential hemodynamic alterations, the cytokines promoting regeneration, and the paracrine communication between hepatocytes and endothelial cells that are involved during liver regeneration were considered [88]. Briefly, spheroids containing primary human hepatocytes and human dermal fibroblasts were resuspended in fibrinogen and thrombin and placed at both sides of the middle channel; this unique channel represented the sinusoid that was embedded with an ECM and where endothelial cells were added. Then, after applying for three days flow alone or flow plus cytokines, the products were collected and analyzed. Interestingly, as a result of applying fluid flow, more than ten cell-derived factors related to liver regeneration were detected; in the presence of cytokines, the hepatocytes also entered into the cell cycle. This approach opens the possibility to explore different combinations of cells, factors, and other conditions, to study the mechanisms affecting liver regeneration in the early stages.

### 3.3. Liver and Hepatocyte Xenotrasplantation

Liver and hepatocyte xenotransplantation have been explored as therapeutical options for liver diseases and a modality to alleviate the organ supply. Pigs, specifically, have a great advantage in their use as cell or whole organ donors. Physiologically they have some similarities to humans, but can be genetically modified to accomplish a more equal genetic background [89,90,91]. One of the major hurdles that has prevented liver xenotransplantation becoming a widely used procedure has been the lack of long-term survival due to hyperacute rejection [92], platelet and red blood cell destruction [93,94,95], and incompatibilities in the coagulation system [96], leading to severe clotting or bleeding and therefore graft loss. There have also been some ethical issues relating to the use of pigs for transplantation purposes, including the risk of zoonotic pathogen transmission such as the porcine endogenous retroviruses (PERV) [97]. However, the recently documented first pig-to-human organ transplant into a patient providing full support and a 2-month survival demonstrated that xenotransplantation is indeed the future of organ transplantation [98]. Therefore, research in this field should continue to seriously investigate the different hurdles that are delaying its establishment as an alternative therapy to overcome the current organ shortage.

In the following section, we will describe some of the advancements in both hepatocyte and liver xenotransplantation, with a special focus on the ability of these xenografts to promote liver function and improve liver regeneration.

#### 3.3.1. Liver Function by Hepatocyte Xenotransplants

Hepatocyte transplantation has already been successfully proven in the clinical setting, especially in children with metabolic hereditary disorders [99] where the partial recovery of the different metabolic functions was documented following transplantation. However, the success of hepatocyte xenotransplantation has so far been only proven experimentally in small and large animal models.

A. Small Animal Models

Xenogeneic porcine hepatocytes have been used with some success in the models of metabolic defects such as severe hypercholesterolemia, where the transplantation of pig hepatocytes led to a decrease in serum cholesterol that lasted for 100 days [100]. Pig hepatocytes have also been transplanted in rats and mice with acute or chronic liver failure chemically induced where an improvement in metabolic function and survival is very well documented [101,102,103]. In surgical models of extended hepatectomy, the benefit of hepatocyte xenotransplantation has also been demonstrated, where hepatocytes were shown to be engrafted in the spleen of the animals and to increase survival and promote liver regeneration [104]. Following transplantation, porcine hepatocytes engraft in different locations, including the spleen and the liver, and demonstrate functionality such as the production of albumin [105,106]. As with other cell sources, fresh hepatocytes are preferred over cryopreserved ones, due to a compromised functionality following cryopreservation [105]. Unfortunately, as with any xenotransplant, one of the major issues to be overcome is rejection; therefore, several strategies to avoid hepatocyte loss have been attempted, including the encapsulation of porcine hepatocytes [107,108]. Their function has been tested in different acute liver failure models, where hepatocytes have been predominantly placed in the peritoneal cavity [109,110]. Comparisons between fresh and cryopreserved encapsulated hepatocytes have also been performed [109,110]. Hepatocytes retrieved at different time points after transplantation have demonstrated in some instances a preserved morphological and ultrastructural appearance, indicating their ability to survive despite not being in the natural liver microenvironment. However, importantly, this shows their ability to overcome rejection by the immunological system.

Even in the case of syngeneic hepatocytes, many cells lost following transplantation are immediately lodged within the portal tracts [111], and become a target of innate immune cells such as macrophages. In the case of hepatocyte xenotransplantation as shown in pigs, this is an even bigger problem since there are several receptor incompatibilities including the very well-known CD47 receptor known as the self-receptor [112,113], and its ligand SIRP-alpha (known as the eat-me signal) [113]. Experimental models using CD47KO hepatocytes to mimic a xenogeneic hepatocyte have demonstrated that they are cleared by innate immune cells due to this receptor incompatibility [114], thus representing an additional barrier to hepatocyte xenotransplantation.

B. Large Animal Models

Large preclinical animal models are the ideal candidates to test a more clinical scenario and the potential of hepatocyte xenotransplantation.

Thanks to the use of these models, additional information regarding the number of cells that would be required to achieve clinical improvement, the potential strategies to be used to increase engraftment, and the rate of cell infusion, among others, has been refined [115,116]. One of the first and few demonstrations of the potential of the pig hepatocytes in preclinical models was that performed by Nagata et al., in 2007. In these experiments, porcine hepatocytes were transplanted into the spleens of cynomolgus monkeys using conventional immunosuppression in order to assess their survival and engraftment ability [116]. By tracking porcine albumin, the authors were able to assess that transplanted hepatocytes survived from 25 to 80 days. Engraftment was confirmed in the spleen of these animals 40 days after transplantation [116]. In large animal models, work has also largely focused on the use of encapsulated hepatocytes injected into the peritoneal cavity for several models of acute liver failure [115]. In these studies, it was demonstrated that encapsulated hepatocytes were able to promote a complete recovery of liver function in some of the animals tested when compared to the animals without transplantation who succumbed to fulminant liver failure [115].

Overall, all the experimental models performed so far, whether in small or in large animals, have attempted to provide additional hepatocyte mass so the liver can recover from the insult and regenerate. Thus, the transplantation of xenogeneic hepatocytes provides an alternative therapy that, although not clinically proven, has enormous potential.

#### 3.3.2. Liver Function by Liver Xenotransplants

While hepatocyte xenotransplantation has not been tested clinically, pig liver xenotransplantation has shown some clinical success that dates to 1993, when a pig liver was placed heterotopically in a patient with autoimmune hepatitis and grade III encephalopathy [117]. The organ functioned and provided some metabolic support for just a few hours [117]. Although the organ was rejected, it demonstrated the clinical feasibility of liver xenotransplantation. Since then, several attempts including the use of transgenic pigs for improving the outcome of pig liver xenotransplantation have been performed using preclinical animal models. Specifically, the modification of several genes aiming at decreasing the hyperacute and acute rejection of the organs and genes related to organ growth has been performed.

The very first pig liver transplants performed in non-human primates in the early 1990s were done using WT pigs, and the post-transplant survival achieved was very limited, ranging from 2 up to 84 h [118,119,120]. Despite the use of immunosuppression, the livers were immediately rejected. In the 2000s, the use of pigs genetically engineered for the h-DAF or CD55 complement regulatory protein improved survival up to 8 days. During these experiments, liver support was documented with special emphasis on the production of coagulation proteins, one of the main synthetic functions of the liver [121]. Bilirubin unfortunately increased over time, reaching levels as high as 18.9 mg/dL towards the end of the study in some animals. Overall, these experimental sets of animals showed that these organs could provide hepatic support to maintain survival up to 8 days [121]. A few years later, additional genes were modified in pig livers that allowed a similar survival ranging from 4 to 9 days [93,122]. The fact that the animals were alive for all those days provides evidence of a somewhat adequate liver function despite the inherent complications associated with coagulation issues and thrombocytopenia due to physiological incompatibilities [93,96,122]. More recently, it was discovered in 2016 that the addition of different coagulation factors, either in bolus or in a continuous infusion, led to an improvement in transfusion requirements and in the development of thrombotic microangiopathy [96]. This report set the stage for subsequent studies where modifications of the infusion rate, dose, and type of coagulation factor, in combination with different immunosuppression regimens, led to the survival of two pig liver recipients for almost a month [123,124].

Pig liver xenotransplantation has also been demonstrated to provide beneficial effects for liver regeneration. For example, the transplantation of an auxiliary xenogeneic pig liver to a model of 90% hepatectomy (Hx) provided a survival benefit, compared to animals subjected to Hx alone. In addition, the resection was able to provide regeneration stimuli including IL-6, which is required as a priming agent for hepatocytes, as demonstrated by the Ki67+ hepatocytes in both the native remnant liver and the transplanted XHALT (Xenogeneic heterotopic auxiliary liver transplantation) [125]. As a result of liver regeneration in both organs, one of the animals survived up to 11 days with the XHALT, while demonstrating fewer coagulation issues, and good pig protein expression [125]. The animals with only Hx succumbed earlier due to severe liver failure. These experiments provide us with additional information regarding the ability of porcine liver transplantation to be used as a bridge while the native liver regenerates, demonstrating an excellent resource that deserves further research efforts.

## 4. Current and Future Ethical Challenges

A controversial topic comprises the ethical issues involved not only in the clinical practice of transplanting human organs, but in technologies focused on relieving the organ shortage available for transplants. Here, we present a brief summary of considerations put to discussion for almost 70 years, related to the limits of “doing good” by avoiding harm to others.

Regarding living donor organ donation, the safety of healthy people is a big concern where a balance between risk to the donor and the high possibility of a successful transplant must be taken into account [126]. Other topics under discussion are: if the altruist donor freely makes his/her decision; the possibility of receiving a reimbursement; or if it is acceptable to use hepatitis C virus (HCV)-infected organs for uninfected recipients [127,128].

Although living donors can donate part of their liver, the shortage of organs is still a big concern. Xenotransplantation as an alternative, but brings new challenges. Clearly, this option is even more risky than orthotopic transplantation due to the high possibility of rejection and the potential emergence of a new virus and even worse, of a pandemic. However, the use of genetically modified animals in the attempt to reduce rejection rates is not exempt from ethical discussion [129,130]. In order to avoid rejection, it is possible to develop a “humanized organ” inside animals for later transplantation. In this case, the ethical concerns refer to the possibility, quite unlikely, of introducing human DNA into an animal embryo, and thereby compromising human dignity.

An emerging ethical concern while xenotransplantation is under experimental research is that once this procedure is established, the production of animals for this purpose, basically pigs, will be discussed in the context of life-saving benefits versus the use of animals for meat consumption. Undoubtedly, many ethical issues will be raised in the process of minimizing the risk of spreading zoonosis to humans, because everyone close to the recipient may well be subject to long-term observation, affecting their rights to privacy and confidentiality.

Based on all the aforementioned aspects, it should be a priority to encourage social change and health policies to prevent the pathologies demanding liver transplants, to promote a donation culture, and to improve useful methods such as 3D bioprinting [131,132,133].

## 5. Conclusions

Science can be conceived as an accumulated knowledge that is constantly updated by new findings improving the understanding of multiple phenomena. Through the years, it has been clearly shown that interdisciplinary work is a fundamental strength for the progress of science. This point of view is supported in this review, where we have compiled important insights of the basic research regarding liver regeneration that have eventually been applied to improve quality of life and human health. It is important to be mindful that research performed using animal models or in vitro techniques must be carried out under strict rules of respect.

As new knowledge and applications become established, new challenges related to social, ethical, and medical issues will certainly emerge. Again, it is up to all the participants involved in this field of study to find the proper path to carry on within this journey of discovery.

## Data Availability

Not applicable.
